# Supplementary material for: Postlarval Shrimp-Associated Microbiota and Underlying Ecological Processes over AHPND Progression
Source: Microorganisms. 2025 Mar 24;13(4):720. doi: 10.3390/microorganisms13040720 (PMC12029802; doi:10.3390/microorganisms13040720)

Supplemental data summary

Postlarval Shrimp-Associated Microbiota and Underlying Ecological Processes over AHPND Progression

Zhongjiang Zhou [1,2], Jiaqi Lu [1,2], Pingping Zhan [2,*] and Jinbo Xiong [1,2,*]

1 State Key Laboratory for the Quality and Safety of Agro-Products, School of Marine Sciences, Ningbo University, Ningbo 315211, China;

2 Key Laboratory of Aquacultural Biotechnology, Ministry of Education, School of Marine Sciences, Ningbo University, Ningbo 315211, China

***Corresponding authors**

Pingping Zhan, E-mail: zhanpingping@nbu.edu.cn

Jinbo Xiong, E-mail: xiongjinbo@nbu.edu.cn

Table S1 Experimental design and sampling schedule of our study.

Health status	Tank	11 dph	15 dph	18 dph	21 dph	Survival rate on 23 dph (%)
Health	1	×	H15-1	H18-1	H21-1	83.5
	2	H11-2	H15-2	H18-2	×	71.2
	3	H11-3	H15-3	H18-3	H21-3	61.6
	4	H11-4	H15-4	H18-4	H21-4	61.4
	5	×	×	H18-5	H21-5	68.4
	6	×	H15-6	H18-6	×	68.1
	7	H11-7	H15-7	H18-7	H21-7	60.2
AHPND	8	A11-1	A15-1	A18-1	A21-1	25
	9	A11-2	A15-2	A18-2	×	5
	10	×	A15-3	A18-3	×	5
	11	A11-4	A15-4	A18-4	A21-4	25
	12	A11-5	A15-5	A18-5	A21-5	20
	13	A11-6	A15-6	A18-6	A21-6	25
	14	A11-7	A15-7	A18-7	A21-7	25

×: Samples did not pass the DNA quality control; AHPND: Acute Hepatopancreatic Necrosis Disease; dph: days post hatching.

Table S2 Comparing relative abundance of the 10 most abundant genera between AHPND infected shrimp and matched controls using unpaired t test. *: $p < 0.05$; **: $p < 0.01$.

	11 dph (days post hatching)		15 dph		18 dph		21 dph	
	Health	AHPND	Health	AHPND	Health	AHPND	Health	AHPND
Vibrio	25.37 ± 19.33	11.02 ± 6.05	13.6 ± 16.49	8.1 ± 7.48	5.67 ± 1.78	11.38 ± 8.58	26.49 ± 17.11	21.81 ± 14.54
Nautella	4.39 ± 1.36	14.86 ± 8.56	6.26 ± 4.34	17.88 ± 7.29*	9.8 ± 5.37	13.26 ± 8.47	5.22 ± 2.29	7.17 ± 1.61
Donghicola	6.16 ± 2.44	9.01 ± 5.45	2.91 ± 0.61	5.46 ± 2.59	4.29 ± 1.48	4.15 ± 2.04	4.38 ± 3.29	6.88 ± 5.5
Ruegeria	3.41 ± 1.54	6.45 ± 6.53	5.45 ± 2.07	4.28 ± 2.03	5.38 ± 1.93	4.43 ± 1.63	4.75 ± 1.72	5.21 ± 1.73
Bacillus	11.26 ± 4.27*	1.67 ± 0.3	10.15 ± 4.7**	2.38 ± 1.3	5.3 ± 1.36**	1.15 ± 0.37	4.19 ± 2.48*	1.09 ± 0.61
Gilvibacter	0.42 ± 0.68	17.56 ± 14.31*	0.38 ± 0.4	7.84 ± 5.87*	0.11 ± 0.07	1.53 ± 1.25	0.04 ± 0.04	0.79 ± 0.68
Spongiimonas	0.89 ± 0.65	4.15 ± 2.84	1.84 ± 1.11	4.01 ± 2.17	1.37 ± 0.75	3.72 ± 3.85	0.54 ± 0.32	1.71 ± 0.74*
Maliponia	0.64 ± 0.45	0.86 ± 0.45	1.16 ± 0.54	2.09 ± 2.53	1.9 ± 1.38	3.94 ± 4.27	3.49 ± 3.17	1.8 ± 0.64
Yangia	1.14 ± 0.63	0.82 ± 0.73	2.01 ± 0.6*	0.41 ± 0.32	3.65 ± 3.97	0.26 ± 0.1	5.07 ± 7.31	0.7 ± 0.56
Leisingera	1.33 ± 1.01	1.73 ± 1.61	1.93 ± 1.18	0.64 ± 0.29	2.28 ± 1.95	0.7 ± 0.65	1.63 ± 1.96	0.59 ± 0.53

Table S3 Topological properties of network between healthy and AHPND infected shrimp after excluding the dph-discriminatory ASVs.

	Health	AHPND
Node	180	169
Edge	849	625
Modularity	0.423	0.475
Average degree	9.43	7.39
Average path length	3.36	3.32
Average clustering coefficient	0.409	0.373
Positive/negative association (%)	70.1/29.8	63.8/36.2

Figure S1. The detected copies number of *pirAB* genes in AHPND infected and healthy shrimp at each sampling. H: healthy shrimp, A: AHPND infected shrimp. Numbers are days post hatching.

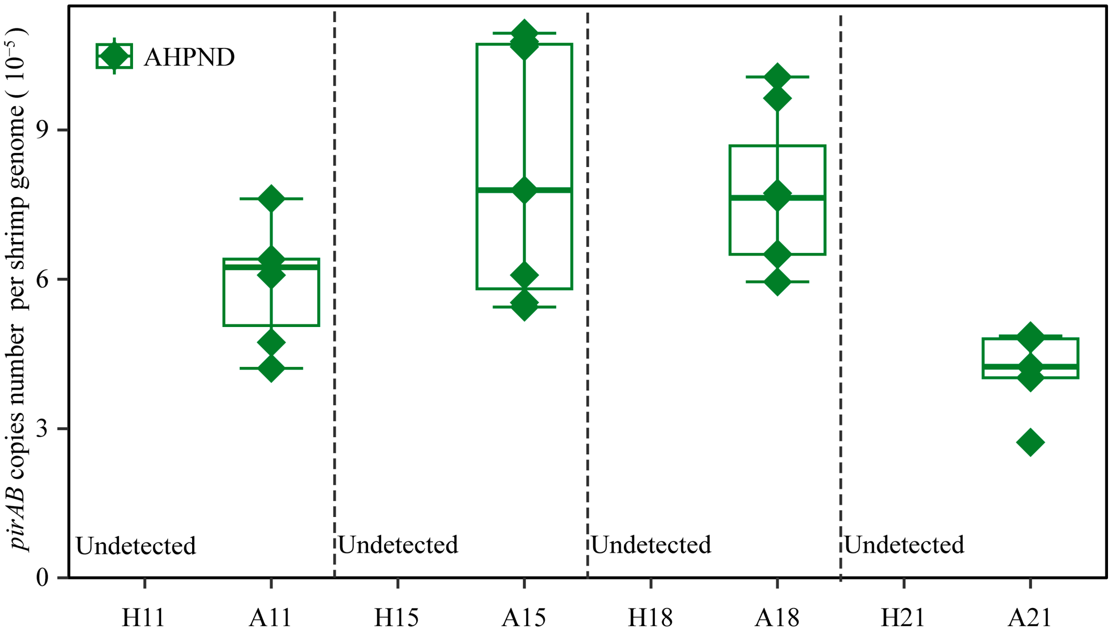

Figure S2. Shrimp survival rate along days post hatching.

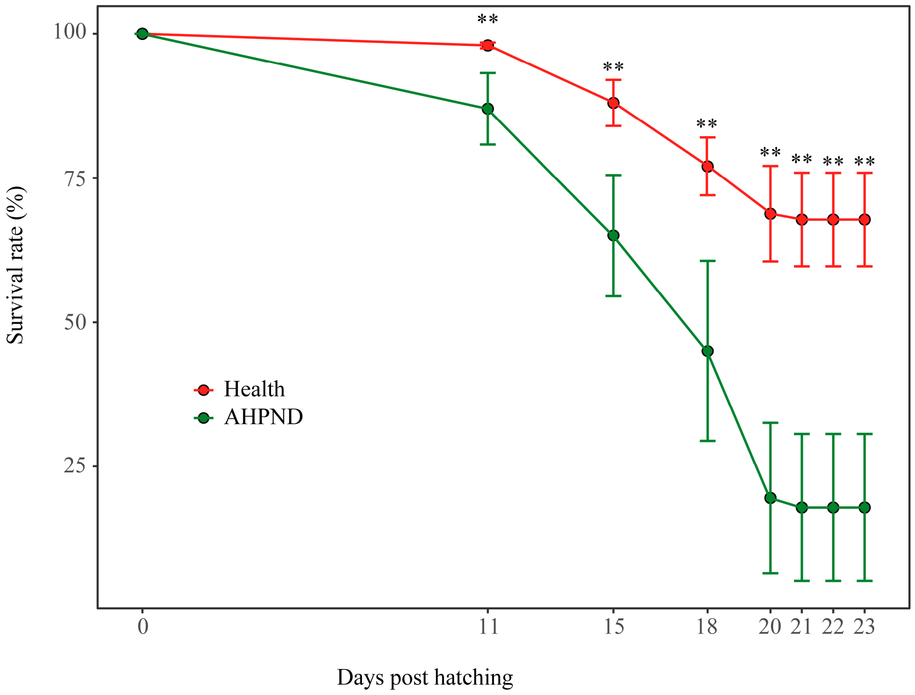

Figure S3. Comparing relative abundance of the dominant bacterial phyla between AHPND infected shrimp and matched controls using unpaired t test. *: $p < 0.05$; **: $p < 0.01$. H: healthy shrimp, A: AHPND infected shrimp. Numbers are days post hatching.

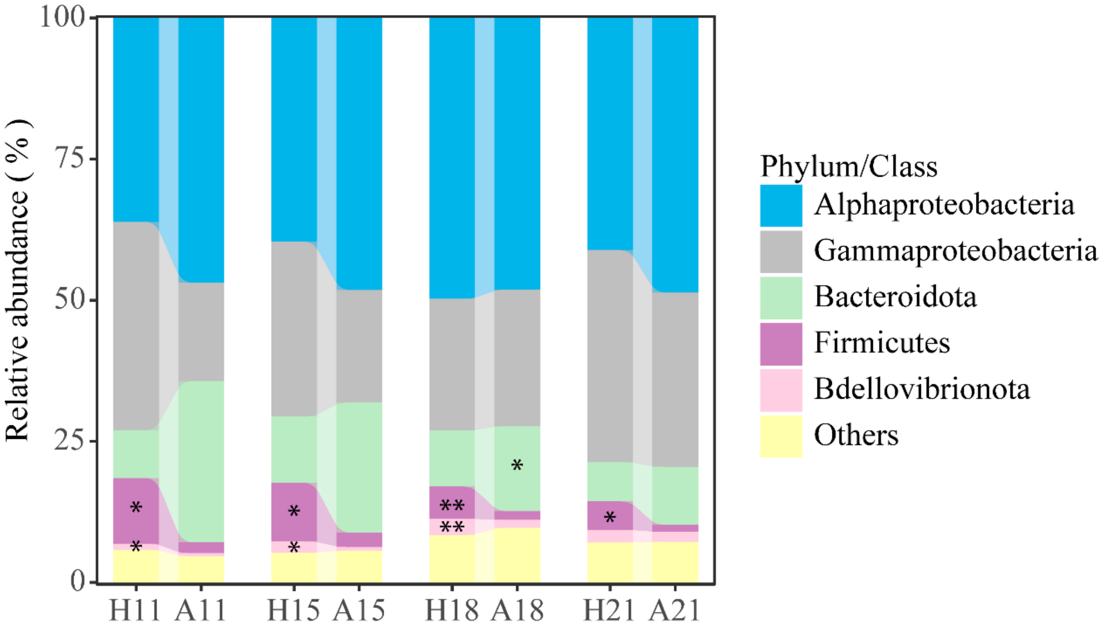

Figure S4. Constrained analysis of principal coordinates revealing the AHPND effect on the larvae associated microbiota on (A) 11, (B) 15, (C) 18, and (D) 21 days post hatching. Significance in community structures between larvae health status was tested using analysis of similarity. H: healthy shrimp, A: AHPND infected shrimp. Numbers are days post hatching.

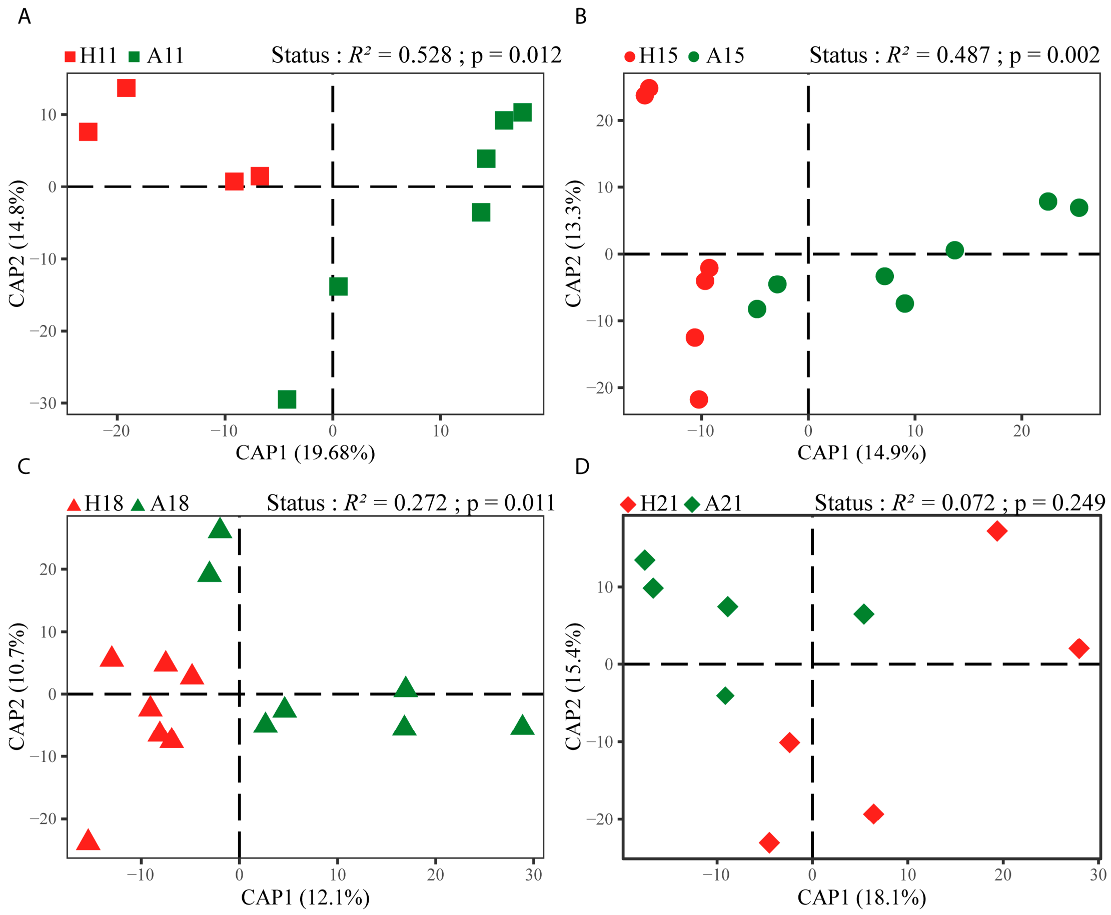

Figure S5. Phylogenetic-bin-based null model analysis indentifies the important Bins that determine the ecological processes governing shrimp associated microbiota.

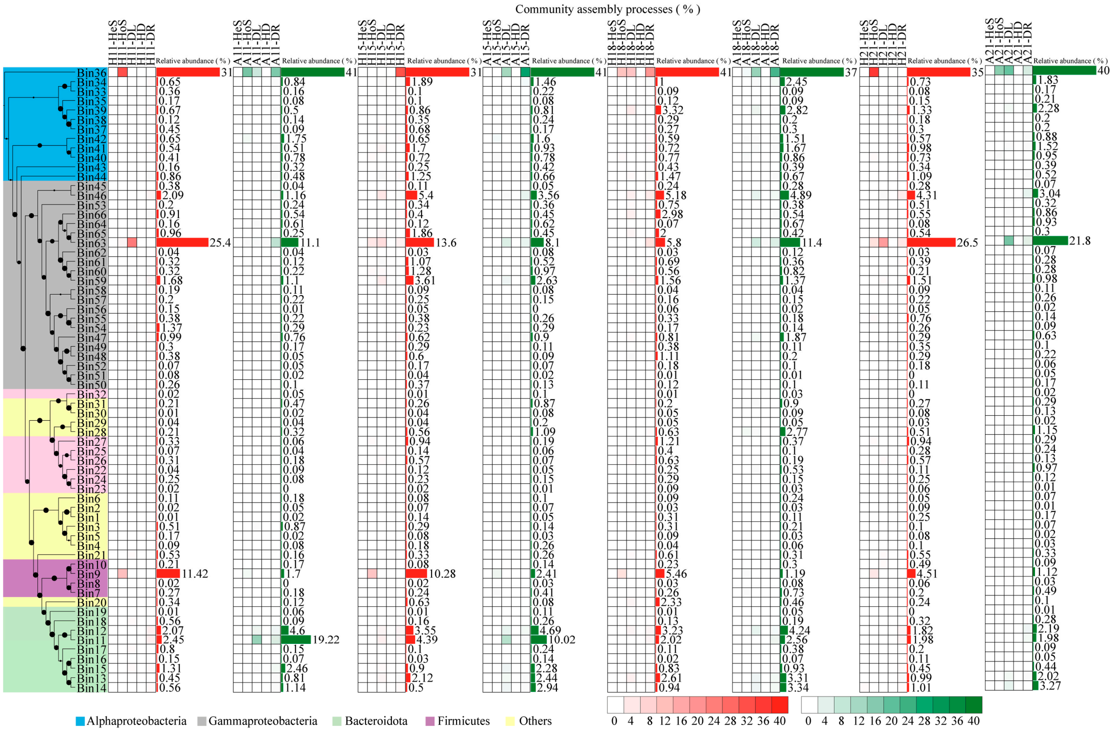

Figure S6. Members of the four important Bins and their distributions across the samples.

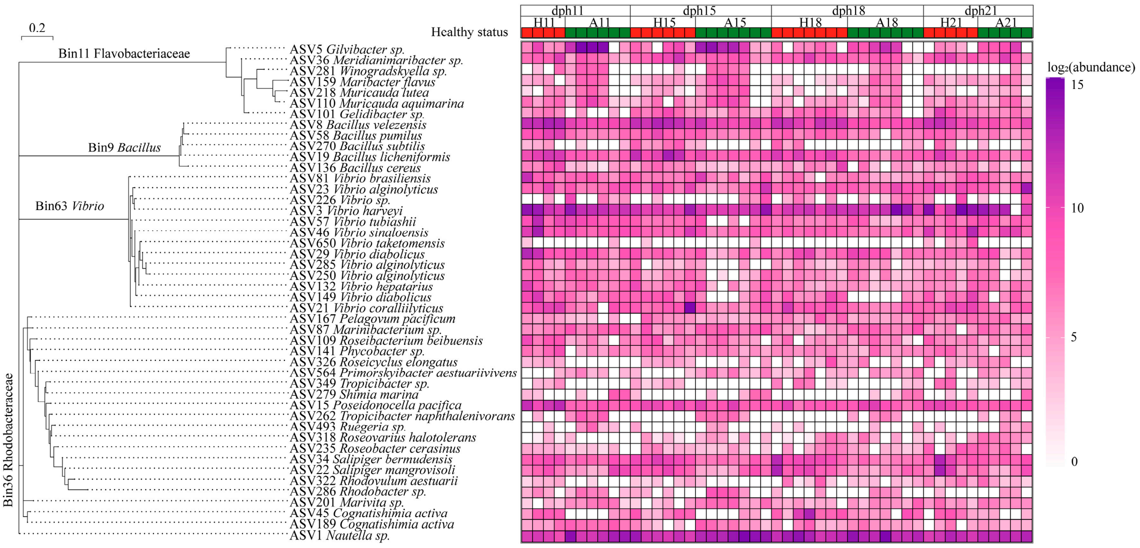

Figure S7. Identification of biomarkers for diagnosing the days post hatching of healthy larvae. (A) The top 19 dph-discriminatory taxa are ascertained using 10-fold cross-validation approach. (B) The variable importance of the 19 dph-discriminatory taxa and correlations between their relative abundances and dph. (C) The consistency between predicted and observed days post hatching of shrimp.

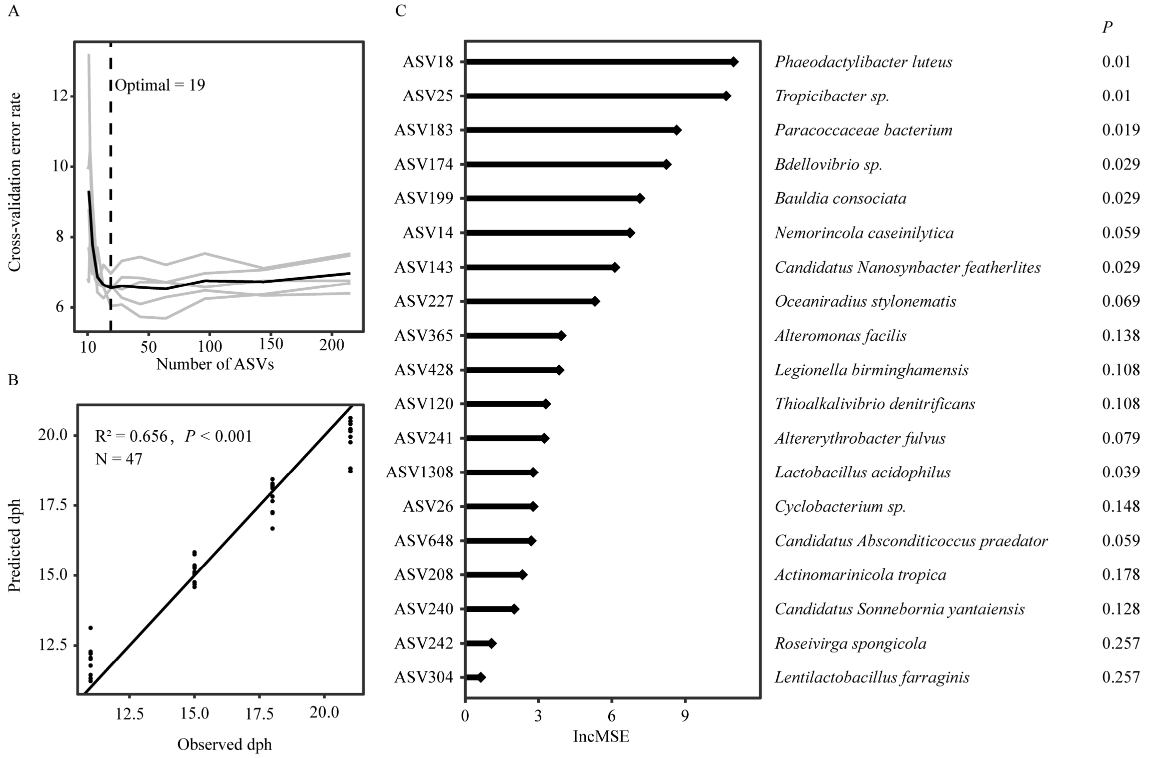

Supplement: Supplementary file 1 [file microorganisms-13-00720-s001.zip › microorganisms-3521038-supplementary.pdf]
